# Preparation of the Pyridinium Salts Differing in the Length of the *N*-Alkyl Substituent

**DOI:** 10.3390/molecules15031967

**Published:** 2010-03-19

**Authors:** Jan Marek, Petr Stodulka, Jiri Cabal, Ondrej Soukup, Miroslav Pohanka, Jan Korabecny, Kamil Musilek, Kamil Kuca

**Affiliations:** 1 Faculty of Military Health Science, University of Defence, Hradec Kralove, 50001, Czech Republic; 2 Vakos XT, Prague, 18600, Czech Republic; 3 Faculty of Pharmacy in Hradec Kralove, Charles University in Prague, Hradec Kralove 50001, Czech Republic

**Keywords:** pyridinium salt, synthesis, micellar catalysts, HPLC, TLC

## Abstract

Quaternary pyridinium salts with chains ranging from C_8_ to C_20_ belong in the large group of cationic surfactants. In this paper, the preparation of such cationic surface active agents based on the pyridinium moiety and differing in the length of the *N*-alkyl chain is described. Additionally, HPLC technique was established to distinguish each prepared pyridinium analogue. This study represents universal method for preparation and identification of quaternary pyridinium detergents.

## 1. Introduction

**Figure 1:**
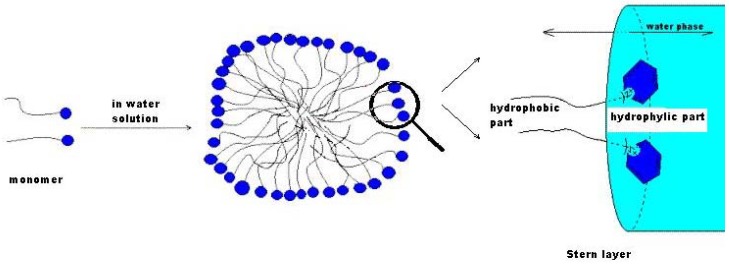
Forming of the micelles.

Cationic surfactants offer a huge amount of applications for industry. They are frequently used in analytical and physical chemistry. Besides, some pyridinium salts are used as disinfectants in various medical applications (e.g., eye drops, solutions, foams). In this field, quaternary pyridinium salts are compounds of high interest, on account of their similarity to benzalkonium salts [1]. Their surfactant characteristics (e.g., critical micellar concentration, surface tension) have already been determined and published everal times [2]. Some pyridinium salts (C_12_ and C_16_) were also used as solubilizers for water insoluble compounds in Analytical Chemistry. Differently, such surfactants were employed in the complexes with dyes to determine the type and concentration of electrolytes by a spectrophotometric method [3]. A method describing spray application of a compound with a C_16_ side chain on the pyridinium ring (cetylpyridinium, CP) for protection of the poultry against bacterial contamination has also been published [4]. Another application of CP consisted in its addition to chewing gums as an anti-plaque agent [5]. The same application may be possible in the dentistry [6]. The surface activity of CP predicted its ability to form the micelles (Figure 1). This property was used by method called micellar-enhanced ultrafiltration that was applied for the removal of the pollutants (e.g., arsenic, perchlorate) from ground waters and also for the removal of the heavy metals from solutions [7,8]. The usage of pyridinium detergents as carriers improving adsorption of the oligonucleotides at the phospholipid membrane was also described [9,10]. The pyridinium analogues with the alkyl chains longer than C_10_ are known as nicotinic receptor antagonists [11]. Some pyridinium salts bearing shorter alkyl chains were prepared and tested as cholinesterase inhibitors [12].

Preparation of the different pyridinium salts was already described before [13,14]. However, there has been no description of the synthesis of the whole series of such salts differing in the alkyl chains (C_8_ to C_20_). Formerly, a similar method for preparation of the benzalkonium salts was reported [15]. Consequently, a universal method for preparation of monoquaternary pyridinium salts with chain substituents was developed. In this study, this method was successfully applied for the preparation of the described pyridinium salts.

## 2. Results and Discussion

The results regarding the synthesis and analysis of the newly prepared compounds are summarized in Table 1. Yield, melting point and HPLC retention time were determined. The preparation of salts with C_12_-C_20_ alkyl chains was found to be easier than in the case of C_8_-C_10_ ones and the final yields were higher. White crystaline products were obtained. Satisfactory purity was obtained after one (C_12_-C_16_) or several crystallizations (C_8_-C_10_, C_18_-C_20_) from ether suspensions. The compounds with C_18_-C_20_ alkyl chains were contaminated with starting material that was detected by TLC. Hence, they were crystallized from acetone. There were also some difficulties in the preparation of C_8_-C_10_ pyridinium salts. These compounds were crystallized several times from ether before the crystals were formed. After all these crystallizations, the yield was dramatically decreased.

**Figure 2:**
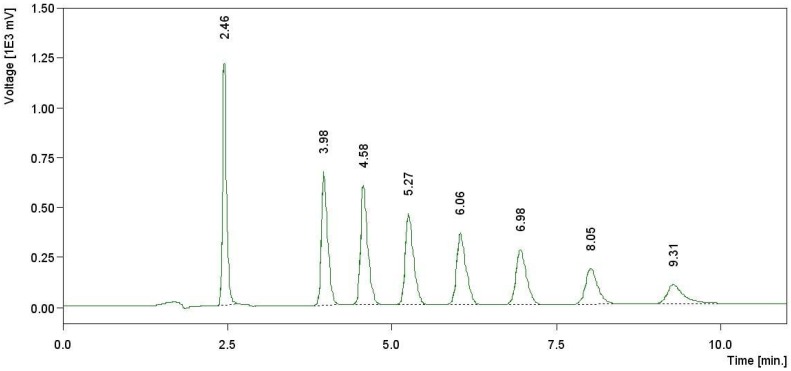
HPLC chromatogram of the prepared pyridinium series mixture.

Additionally, the HPLC analysis was carried out for individual compounds. A newly developed HPLC method was able to distinguish among all prepared quaternary pyridinium salts (Figure 2). The shortest retention time was found for the C_8_ pyridinium salt. This novel HPLC method could be easily used for characterization of mixtures of pyridinium detergents.

## 3. Experimental 

### 3.1. General

The reactions were monitored by TLC (Kieselgel Merck; mobile phase chloroform/methanol 100/1; detection UV 254, Dragendorff reagent). The yields (%) and melting points (Boetius, m.p. were uncorrected) are summarized in Table 1. ^1^H-NMR spectra characterizing individual compounds were recorded in DMSO-d_6_ on a Varian Gemini 300 instrument.

### 3.2. Synthesis

**Scheme 1:**
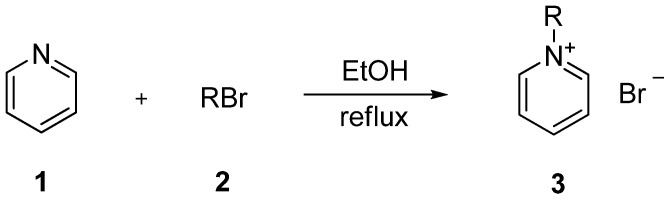
Preparation of pyridinium salts.

A universal method for the preparation of monoquaternary pyridinium salts **3-9** was developed (Scheme 1): pure pyridine (**1**; 1 eq) in dry ethanol was mixed with 1-bromoalkane (**2**; 1.4 eq). The mixture was refluxed for 40 hours. The solution was evaporated under reduced pressure and the crude oily product was crystallized from ether, filtered under reduced pressure, washed with ether and allowed to dry at r.t.. 

*N-Octylpyridinium bromide* (**3**): ^1^H-NMR ppm 9.12 (d, *J =* 5.7 Hz, 2H), 8.61 (t, *J =* 7.6 Hz, 1H), 8.22-8.11 (m, 2H), 4.58 (t, *J =* 7.5 Hz, 2H), 1.98-1.80 (m, 2H), 1.22 (m, 12H), 0.82 (t, *J =* 6.6 Hz, 3H).

*N-Decylpyridinium bromide* (**4**): ^1^H-NMR ppm 9.14 (d, *J =* 5.63 Hz. 2H), 8.61 (t, *J =* 7.58 Hz, 1H), 8.22-8.11 (m, 2H), 4.60 (t, *J =* 7.46 Hz, 2H), 2.01-1.80 (m, 2H), 1.16 (m, 16H), 0.83 (t, *J =* 6.64 Hz, 3H).

*N-Dodecylpyridinium bromide* (**5**): ^1^H-NMR ppm 9.14 (d, *J =* 5.6 Hz. 2H), 8.61 (t, *J =* 7.8 Hz, 1H), 8.22-8.11 (m, 2H), 4.61 (t, *J =* 7.5 Hz, 2H), 1.98-1.80 (m, 2H), 1.23 (m, 20H), 0.79 (t, *J =* 6.3 Hz, 3H).

*N-Tetradecylpyridinium bromide* (**6**): ^1^H-NMR ppm 9.14 (d, *J =* 5.6 Hz, 2H), 8.60 (t, *J =* 7.8 Hz, 1H), 8.22-8.11 (m, 2H), 4.61 (t, *J =* 7. 5 Hz, 2H), 1.98-1.80 (m, 2H), 1.23 (m, 24H), 0.83 (t, *J =* 6.6 Hz, 3H).

*N-Hexadecylpyridinium bromide* (**7**): ^1^H-NMR ppm 9.13 (d, *J =* 5.7 Hz, 2H), 8.61 (s, 1H), 8.22-8.11 (m, 2H), 4.61 (t, *J =* 7.4 Hz, 2H), 2.03-1.80 (m, 2H), 1.21 (m, 28H), 0.83 (t, *J =* 6.5 Hz, 3H).

*N-Octadecylpyridinium bromide* (**8**): ^1^H-NMR ppm 9.12 (d, *J =* 5.4 Hz, 2H), 8.61 (s, 1H), 8.22-8.11 (m,, 2H), 4.60 (t, *J =* 7.3 Hz, 2H) 1.89-1.80 (m, 2H), 1.22 (m, 32H) 0.82 (d, *J =* 6.3 Hz. 3H).

*N-Eicosylpyridinium bromide* (**9**): ^1^H-NMR ppm 9.14 (d, *J =* 5.63 Hz, 2H), 8.60 (t, *J =* 7.8 Hz, 1H), 8.23-8.11 (m, 2H), 4.61 (t, *J =* 7.5 Hz, 2H), 1.98-1.80 (m, 2H), 1.23 (m. 36H), 0.83 (t. *J =* 6.6 Hz, 3H).

### 3.3. HPLC Analysis

A suitable method for the characterization of the newly prepared compounds in the mixture was developed using HPLC. The HPLC system consisted of a P200 gradient pump (Spectra-Physics Analytical, Fremont, CA, USA), a 7125 injection valve – 10 µL loop (Rheodyne, Cotati, CA, USA), an UV1000 detector (Spectra-Physics Analytical) and a CSW Chromatography Station 1.5 software (DataApex, Praha, Czech Republic). A 250 × 4.6 mm I.D. Waters Spherisorb cyano (5 µm) column was used for analysis (Supelco Inc., Bellefonte, WA, USA). The mobile phase consists of 45% acetonitrile and 55% water and was prepared as 0.1 M sodium acetate solution. Finally the pH was adjusted via acetic acid to 5.0. The column was rinsed isocratically with a flow-rate of 1 mL/min. The absorbance was measured at 257 nm.

## 4. Conclusions

The preparation of pyridinium salts with C_8_-C_20_ *N*-alkyl chains was described. Such preparation might be used not only under laboratory conditions, but also for semi-industrial purposes. It is anticipated that the synthesis wil prove very useful for the next effort of preparing micelle forming compounds. The developed HPLC experimental protocol was found valuable for the estimation of the purity of the newly prepared compounds. 

## Figures and Tables

**Table 1:** Yield, melting point and retention time of prepared pyridinium salts.

Compound	Side alkylating chain (R)	Yield (%)	m.p. (°C)	HPLC R_t_ (min)
**3**	C_8_	13	28–30	3.98
**4**	C_10_	43	30–32	4.58
**5**	C_12_	82	73–75	5.27
**6**	C_14_	76	58–60	6.06
**7**	C_16_	99	63	6.98
**8**	C_18_	95	65–67	8.05
**9**	C_20_	79	73–75	9.31
